# Bystander Memory T Cells and IMiD/Checkpoint Therapy in Multiple Myeloma: A Dangerous Tango?

**DOI:** 10.3389/fimmu.2021.636375

**Published:** 2021-02-15

**Authors:** Anne Marit Sponaas, Anders Waage, Esten N. Vandsemb, Kristine Misund, Magne Børset, Anders Sundan, Tobias Schmidt Slørdahl, Therese Standal

**Affiliations:** ^1^Department of Clinical and Molecular Medicine, Center for Myeloma Research, Faculty of Medicine and Health Sciences, Norwegian University of Science and Technology (NTNU), Trondheim, Norway; ^2^Department of Hematology, St.Olavs Hospital, Trondheim, Norway; ^3^Department of Immunology and Transfusion Medicine, St.Olavs Hospital, Trondheim, Norway; ^4^Department of Clinical and Molecular Medicine, Center of Molecular Inflammation Research, Faculty of Medicine and Health Sciences, Norwegian University of Science and Technology (NTNU), Trondheim, Norway

**Keywords:** multiple myeloma, T cells, checkpoint therapy, immunomodulating drugs, virus

## Abstract

In this review article we discuss the role of the memory T cells in multiple myeloma (MM) and how they may influence immune responses in patients that received immunomodulating drugs and check point therapy.

## Introduction

Checkpoint therapies with anti-PD1/PDL1 have been successful in malignant melanoma and lung cancer. In MM however, monotherapies with-anti PD1 were not effective. Initially, combining anti PD1 with immunomodulating drugs (IMiDs) demonstrated acceptable toxicity and objective overall response in 44% of patients in a phase I/II clinical study (1). However, when tested in 2 phase III clinical trials, Keynote 183 and 185, there were more deaths in the checkpoint arm and the studies were terminated (2, 3). This led to the termination of many other clinical studies involving PD1/PDL1. Understandably, there has been some reluctance in revisiting PD1/PDL1 therapy in MM. However, we do not yet fully understand why PD1/PDL1 treatment failed and why some patients suffered fatal side effects. In this review we will discuss how T cells could be involved in adverse effects and failure of PD1 therapy.

## Immune Responses in MM Patients

T cells control and remove MM after recognition of tumor specific or tumor associated antigen (TAA). This happens directly by CD8 T cells killing the tumor cells or indirectly by activating NK cells or macrophages after cytokine release such as IFNg (4). Conversion from MGUS and Smoldering Multiple Myeloma (SMM) to MM is characterized by changes in the immune cells in the bone marrow (BM). This includes an alteration in the myeloid cell populations in the bone marrow such as a reduction in the ability of DC to stimulate T cells, switch from M1 to M2 macrophages and increase in immunosuppressive Myeloid Derived Suppressor Cells (MDSC) reviewed in Guillerey et al. (4). There is also evidence that cells in the BM interact to induce an immunosuppressive environment, for example BM-derived IL18 will generate MDSC that suppress CD8 T cell responses in mice and was associated with poor survival in humans (5). There is also a change in T cell phenotype. This is associated with a decrease of T effector functions such as cytotoxicity and IFNγ and an increase in surface expression of many exhaustion markers including PD1, TIGIT, LAG3 and changes in transcription factors such as Eomes and TCF1 (6–8).Interestingly, anti TIGIT antibodies reactivate exhausted T cells and reduce tumor load in a mouse model of MM (8).

Expansion of CD8 T cell clones have been observed in MM patients and were associated with improved survival, and interestingly, these clones expanded after treatment with IMiDs (9) Myeloma patients also have senescent and senescent like KRLG1+, CD57+, CD160+, CD28-, CD8 T cells. Whilst the senescent-like T cells have normal telomere-length and can produce some inflammatory cytokines, the senescent T cells have short telomers and are nonfunctional (10). These cells do not express PD1 and are unable to respond to anti PD1 check point therapy and this was suggested to be a reason for the failure of response to anti PD1 in MM patients (10–12). There is an increase in Treg cells in the tumor microenvironment (TME) in the bone marrow (BM) of patients which is associated with poor outcome and early relapse (13–15). Many of them have an activated phenotype and are directly promoting MM cell growth (14, 15). However, MM is also characterized by an inflammatory T cell response with elevated levels of Th17 (16), and IL32 activity (17, 18), and increased numbers of T effector cells (TEMRAs) (17, 19). Thus, the immune milieu in the BM in myeloma patients can both be inflammatory and immune suppressive, leading to thwarted immune responses to the tumor and pathogens, yet promoting an inflammatory environment that could generate osteoclast activity and bone lesions (20–22). Hypogammaglobulinemia is associated with MM and increases the susceptibility to infections, as will immune suppression and hyper-inflammatory environment. Reduced response to infections contributes to significant comorbidities for MM patients who has 7-fold increase in contracting bacterial and a 10-fold increase in developing viral infections compared with age-matched controls (23). Modern treatment can also modulate immune cells and new therapies have led to increased rates of bacterial, viral and fungal infections (24). However, most MM patients can mount a T cell response that generate some protection against infections.

## T Cells in MM Bone Marrow

There are different populations of T memory cells (Tmem), some are circulating and others resident in the TME. These are characterized by different surface markers and transcription factors. In this paper we are focusing on CD8 T cells as these are important for anti-tumor activity. This is not to say that CD4 T cells are unimportant. Although reduced in some MM patients, CD8+ T central memory (Tcm) (CD45RA−,CD45RO+,CCR7+), T effector memory (Tem) (CD45RA+,CD45RO+,CCR7−) as well as T resident memory (Trm) (CD45RA–,CD45RO+,CD103+,CD69+) are found in the bone marrow (17, 19, 25). In addition to the markers above, memory cells also express certain transcription factors whose expression may vary according to the degree of activation and the milieu in the TME. For example, T mem cells from MGUS patients will have higher expression of stemness marker TCF1 and less Eomes and Tbet compared with T mem from myeloma patients (17). Some of these cells recognize known tumor antigens such as Germline-Associated Antigens (GAAs,) tumor associated antigens (TAAs) or neo antigens (26–29). T memory cells from bone marrow of MM were also found to kill autologous myeloma cells (25). However, common to what is found in solid tumors where neoantigen-specific T cells contribute <0.5% of CD8 T cells (30), the proportion of tumor-specific or TAA T cells in the bone marrow may also be relatively small. Indeed, memory T cells with non-tumor-specificities are found in the BM TME (19). This is not surprising as the human bone marrow is a reservoir for memory T cells against previous infections (31).

## Memory T Cells Against Infectious Agents

Long-term immunity to pathogens is maintained by circulating and resident T and B cells. With the median time of diagnosis at 70 years, MM is more common in elderly people. People above the age of 60 have a reduced bone marrow output with fewer and less efficient hematopoietic stem cells (HSC) compared with younger people. Hematopoiesis is also skewed in a myeloid direction with lower lymphoid output as well as reduced TCR diversities. Many elderly people will also have more innate and adaptive immune cells with inflammatory phenotypes (32). A large proportion of our T memory cells will be against common latent and recurrent viral infections. About 90–95% of people world-wide have been infected with and carry the Epstein-Barr virus (EBV) (33). Likewise, up to 90% of humans are seropositive for Cytomegalo virus (CMV) (34). Other latent virus infections like Herpes simplex virus (HSV-1) also generate long-lived T memory responses.

**CMV** has lytic and latent stages. Once the host is infected, CMV will remain causing recurrent latency and reactivation (34). Healthy individuals do not have long-term problems, but CMV can have serious consequences for immunocompromised patients. Infection generates high frequencies of CMV-specific CD4 and CD8 T memory cells. Control of CMV requires an active adaptive immune response where the CD8 T cells are crucial. The pool of CMV-specific memory T cells increase with age and can be up to 40% of the total memory T cell population. It is often oligoclonal and characterized by the presence of many senescent CD28–CD57+ and terminally differentiated CD45RA+CD45RO– TEMRAs with reduced functionality (35). In addition to providing a poor immune response to the CMV itself, the presence of these expanded, often terminally differentiated and senescent T cells could by their large numbers diminish immune responses to pathogens and vaccines (36) or possibly even to cancers (37). In addition, most myeloma patients below the age of 70 will undergo autologous bone marrow transplantations (ASCT) as part of the therapy. This may reactivate CMV infection (38) and thus generate new anti-CMV T cell responses.

Most **EBV** infections are asymptomatic, but they can develop into infectious mononucleosis (IM) that will resolve in adolescents. The infection is first lytic releasing virus particles, and then enters a growth transforming latency program in B cells. The infection is characterized by sequential expression of viral genes recognized by CD8 and CD4 T cells. In some of the latent infected B cells the virus gene expression will be turned off avoiding immune responses. Similar to CMV, EBV can be reactivated at a later stage. The primary symptomatic infection is characterized by a massive expansion where up to 50% of all CD8 T cells are specific to viral proteins (39–41). After acute infection about 2% of the CD8+ memory cell pool will consist of cells specific to lytic antigens, and about 0.5% will recognize latent antigens (41). This relatively high frequency of EBV-specific CD8 memory cells can be found decades after the primary infection (42). Interestingly, the specificity of these virus -specific T cell clones (in terms of CDR sequence of the TCR) is remarkably stable over time including after lymphodepletion with cytostatic drugs (43). EBV specific T cells can also be detected in patients after bone marrow transplantation, including autologous stem cell therapy (ASCT) where they can be transferred during infusion of stem cells (44, 45) and/or generated after reactivation of virus (46).

CD8 T cells against various EBV, CMV, and influenza epitopes were found among TILs in lung and colon cancer biopsies (30, 47). These cells expressed markers of T resident memory cells (Trms—CD45RO+CCR7–CD103+CD69+) as well as PD1. Interestingly, they were more abundant in the tumor than tumor neoantigen- and TAA-specific T cells. The virus-specific CD8 T cells expressed similar levels of activation and inhibitory molecules as the tumor-specific T cells except from CD39 (30). CD39 is an exoenzyme involved in the generation of immunosuppressive adenosine in cancers such as MM (48).

## Bystander T Cells in Cancer and Immunotherapy

Bystander responses take place when T cells are activated independent of their antigen specific TCR or by cross reactive antigens. This can happen several ways:

**Molecular mimicking** of antigenic epitopes can activate T cells with cross-reactive TCRs. This has been described after infections with for example *Streptococcus pneumonia* and *Borrelia burgdorferi* where the pathogen induces T cell responses cross-reactive to self-antigens (49). Such responses have been linked to multiple sclerosis and cardiomyopathy (50, 51).

Allelic exclusion of TCRα chain during VDJ recombination is sometimes incomplete. This can lead to generation of **T cells expressing 2 TCRs** with different specificities. Indeed, up to 33% of human T cells can have 2 functional α chains (52). Activation of T cells with dual receptors has been implicated in several models of autoimmune disease [reviewed in (53)].

**Super-antigens** such as bacterial endotoxins can also non-specifically engage T cells with different Vβ TCRs leading to release of inflammatory cytokines and induction of self-reactivity (54).

Finally, T cells express **innate and innate-like** receptors. TLR2 and TLR4 engagement with lipoproteins or LPS can activate T cells non-specifically (55). TLR2 is expressed on memory T cells and there are reports of TLR2-mediated stimulation of EBV-specific memory cells leading to improvement of the anti-EBV response (56). TLR8, TLR7, and TLR9 have also been detected on T cells and could be involved in generation of bystander effects (57). For example, increased numbers of activated TILs were found in cutaneous melanomas after direct application of the TLR7 agonist imiquimod (58). NKG2D is an activating receptor on NK and T cells. This receptor binds non-classical MHC-like ligands such as MHC class I polypeptide-related sequence A (MICA) and heat shock protein 60 that can stimulate T cells (53). NKG2D activation is augmented in the presence of cytokines such as IL-2, IL-7, and IL-15 (59–61). NKG2D engagement on CD8 memory T cells can also circumvent the need for CD4 T cell help, thus lowering the threshold for CD8 T cell activation.

Activation of bystander T cells will also depend on the presence of factors such as cytokines within the local environment. Memory T cells are more likely to become bystander cells than naïve T cells as they express higher levels of cytokine receptors (62) and can for example respond to elevated levels of IL-12, IL-15, and IL-18 present at high levels particularly during bacterial infections [reviewed in (53)].

## Checkpoint Therapies and Immune-Related Adverse Effects

Check point therapy with anti-CTLA4 will lower the threshold for initiation of immune responses as well as inhibiting Treg activities, and anti-PD1/PDL1 reactivates exhausted immune responses. However, reactivation of exhausted T cells may not be the only way checkpoint therapies work. Clonal expansion of non-exhausted, tumor reactive effector T cells (63) or recruitment and differentiation of stem cell-like memory cells (64) are also responsible for tumor regressions during treatment.

In recent years it has become clear that checkpoint therapies can cause IRAE in some patients. Both anti-CTLA4 (ipilimumab) and anti-PD1 (pembrolizumab, nivolumab) can elicit IRAE, anti-CTLA4 tend to give more severe effects. The most common IRAEs will affect the gastrointestinal tract, liver, endocrine glands and skin leading to for example colitis, thyroiditis, hepatitis and vitiligo. However, although less common, cardiovascular, pulmonary, hematological and nervous system organs can be involved. Some of these IREAs can lead to serious complications and even death. Several cases of fatal fulminant myocarditis with T cell infiltration has been reported in patients treated with checkpoint therapies (65). Indeed, with a relatively high incidence of 0.6–1%, cardiologists recommend a thorough cardiac assessment before commencing treatment (66).

The exact mechanism behind the IRAEs is not fully elucidated, but it is reasonable to hypothesize that activation of quiescent, self-reactive T cells would be involved.

Studies in mouse models have shown that PD1-/- mice develop autoimmunity including cardiomyopathy (67) and anti-PD1/PDL1 treatment can activate anergic auto-immune T cells inducing organ damage (68). Memory T cells reactive to pathogens could also be culprits and be activated as bystander cells to generate IRAEs. Recently, involvement of oligoclonal, cytotoxic, EBV-specific CD4+ memory cells in immune encephalitis in a melanoma patient receiving pembrolizumab was reported (69). The virus was expressed in lymphocytes at the encephalitic site of inflammation. Thus, it is possible that checkpoint therapy induced IREAs are caused by non-specific activation of virus- specific bystander T cells or by activation of virus-specific cells stimulated by reactivated virus.

## IMiDs, Checkpoint Therapy and Multiple Myeloma

IMiDs such as lenalidomide, pomalidomide, and thalidomide are standard treatment for MM at various stages of the disease. They are used alone or in combination with other drugs. IMiDs target cereblon, a component of a ubiquitin ligase complex. This leads to the degradation of transcription factors IKZF1 and IKZF3 inducing myeloma cell death. IMiDs also degrade IKZF1 and IKZF3 in immune cells These transcription factors are important in T cell function. IKZF1 binds the IL-2 gene promoter and represses IL-2 production and proliferation in T cells (70, 71) thus controlling important steps in induction of effective immune responses, regulation of inflammation as well as prevention of autoimmunity. IMiDs also augment TCR signaling *via* CD28 leading to upregulation of Nfkb (72). T cell from MM patients who respond to lenalidomide have more IFNγ, granzyme B and perforin-positive T cells and fewer terminally differentiated T cells (CD45RA+, CD57+) than those refractory to treatment. Furthermore, lenalidomide treatment *in vitro* increases proportion of myeloma antigen- specific BM T cells with a memory phenotype (73, 74). Interestingly, similar to what is seen in T cells treated with lenalidomide, T cells from patients with autoimmune diseases express low levels of IKZF1 (75).

Most patients respond well to IMiDs, but side effects are not uncommon. These are generally not immunological although recurrent bacterial infections due to neutropenia is a common side effect. However, a combination of IMiDs and anti-PD1 could lower the threshold of activation for memory T cells, anergic or tolerant T cells, thus leading to inflammation and organ damage. In Keynote 185, relapsed and refractory MM patients were treated with pembrolizumab, pomalidomide and dexamethasone (3) and in Keynote 183 newly diagnosed MM patients were treated with pembrolizumab, lenalidomide and dexamethasone (2). As with most of the clinical studies with anti PD1/PDL1 [Supplemental Tables 1, 2; (76)] neither studies reported positive effects, but more deaths were observed in the pembrolizumab arm. In Keynote 185 19 deaths were observed in the pembrolizumab arm compared with 9 in the control with HR for OS at 2.06. In Keynote 183 there was 29 deaths in the pembrolizumab arm compared with 21 and a HR for OS of 1.61 at 8.1 month. In both studies, the incidence of serious adverse events (SAE) were higher in the pembrolizumab arm compared with control (Keynote 183 SAE 63 vs. 46%, Keynote 185 AE were 54 vs. 39%). Many of the deaths in both trials were recorded as cardio-respiratory. Indeed, one patient in Keynote 185 died of fulminant myocarditis (77). This patient did not have a previous history of heart disease or autoimmunity but developed myocarditis on day 16 post-treatment with extensive infiltration of Granzyme B positive CD8 T cells in the myocardium. The patient was previously treated for breast cancer with radiotherapy and this could have generated heart antigen specific T memory cells that were activated during treatment with pembrolizumab.

**Figure 1:**
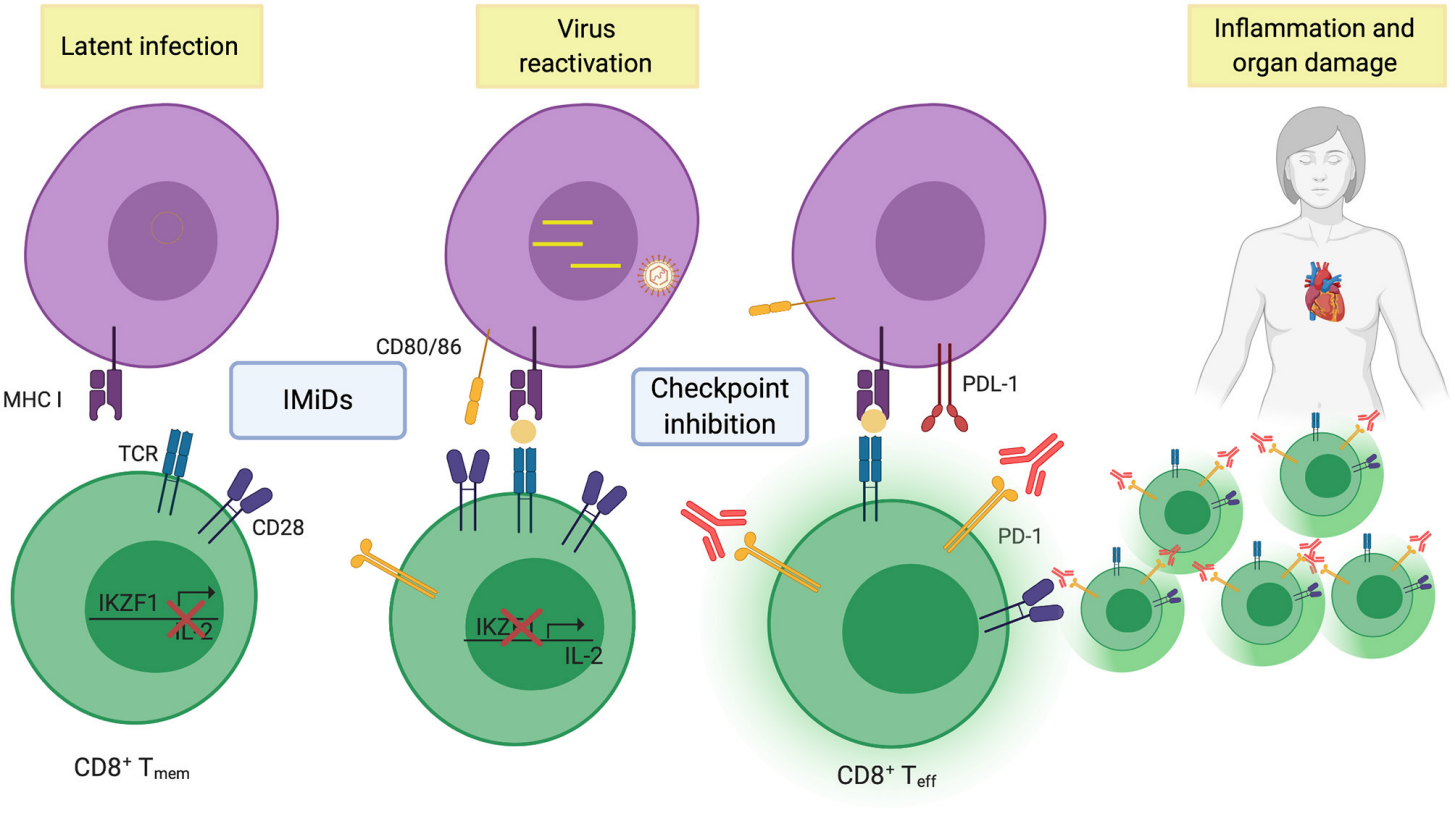
Illustrating re-activation of effector T-cells to viral antigens after combined IMiD and checkpoint therapy. (Created with BioRender.com).

Could activation of virus-specific memory cells also lead to organ damage in the patients treated with IMiDs and checkpoint inhibitors? (as illustrated in Figure 1) Presence of CMV in cardiomyocytes are common in patients with fatal myocarditis (78), and EBV infections have occasionally been associated with myocarditis (79). Similar to solid tumors, EBV-specific PD1+ memory CD8 T cells are found in the bone marrow of MM patients (19) and it is reasonable to speculate that these could be activated during treatment and cause organ damage. Indeed, IMiDs can reactivate latent EBV (80) and anti-PD1 therapy can be used to promote virus clearance in animals with exhausted T cells (81). If this is the case, screening patients for the presence of virus-specific T cells and use of antivirals could prevent IREA. Thus, dissecting T cell responses in bio-banked samples from the clinical trials with IMiDs and anti-PD1 would be very important. Understanding the failure of response to checkpoint therapies in MM and what caused the deaths, would be very useful in developing new, efficient and safe immunotherapies for MM patients.

## Concluding Remarks

T cells are crucial for myeloma patients, in the response to the tumor, in treatment as well as protection against common pathogens. In most adults, the T cell repertoire and the ability of the T cells to respond is shaped by what the immune system has encountered previously. Thus, to develop new types of immunotherapy, it is important to take the presence of tolerant and anergized autoimmune T cells as well as memory T cells to common pathogens into consideration. If we ignore these, we might get some unpleasant surprises.

## Author Contributions

AS, AW, EV, TSl, and TSt: wrote paper. AS, MB, and KM: discussed and edited wrote paper. All authors: contributed to the article and approved the submitted version.

## Conflict of Interest

The authors declare that the research was conducted in the absence of any commercial or financial relationships that could be construed as a potential conflict of interest.
